# Recent Advances of Ocular Drug Delivery Systems: Prominence of Ocular Implants for Chronic Eye Diseases

**DOI:** 10.3390/pharmaceutics15061746

**Published:** 2023-06-15

**Authors:** Mahmoud Mostafa, Adel Al Fatease, Raid G. Alany, Hamdy Abdelkader

**Affiliations:** 1 Department of Pharmaceutics, Faculty of Pharmacy, Minia University, Minya 61519, Egypt; mahmoud_mohamed@mu.edu.eg; 2 Department of Pharmaceutics, College of Pharmacy, King Khalid University, Abha 62223, Saudi Arabia; afatease@kku.edu.sa; 3 School of Pharmacy, Kingston University London, Kingston Upon Tames KT1 2EE, UK; r.alany@kingston.ac.uk; 4 School of Pharmacy, The University of Auckland, Auckland 1010, New Zealand

**Keywords:** chronic eye diseases, inserts, implants, ocular delivery

## Abstract

Chronic ocular diseases can seriously impact the eyes and could potentially result in blindness or serious vision loss. According to the most recent data from the WHO, there are more than 2 billion visually impaired people in the world. Therefore, it is pivotal to develop more sophisticated, long-acting drug delivery systems/devices to treat chronic eye conditions. This review covers several drug delivery nanocarriers that can control chronic eye disorders non-invasively. However, most of the developed nanocarriers are still in preclinical or clinical stages. Long-acting drug delivery systems, such as inserts and implants, constitute the majority of the clinically used methods for the treatment of chronic eye diseases due to their steady state release, persistent therapeutic activity, and ability to bypass most ocular barriers. However, implants are considered invasive drug delivery technologies, especially those that are nonbiodegradable. Furthermore, in vitro characterization approaches, although useful, are limited in mimicking or truly representing the in vivo environment. This review focuses on long-acting drug delivery systems (LADDS), particularly implantable drug delivery systems (IDDS), their formulation, methods of characterization, and clinical application for the treatment of eye diseases.

## 1. Introduction

Over the last three decades, the prevalence of sight-related diseases has received increased attention; this is mainly due to the increasing life expectancy of the global population. There were around 188 million people who had minor vision impairment, 216 million people who had moderate-to-severe sight impairment, and approximately 40 million people who were legally blind [1]. These numbers are only expected to increase over time. The eye has a complicated vital structure with several anatomical and physiological constraints. The anterior part of the eye, which is implicated in refraction and vision, is made up of several ocular tissues, including the cornea, conjunctiva, aqueous humor, iris, ciliary body, and the lens, whereas the back segment of the eye is mostly made up of the vitreous humor, choroid, retina, and posterior sclera. The posterior segment recognizes and transmits light signals though the optic nerve so that the eye can view the outside world. Many chronic eye diseases can affect these specialized ocular tissues.

Common conditions that affect the front of the eye include glaucoma, anterior uveitis, cataracts, and dry eye diseases [2,3], while the conditions that most often affect the back of the eye include AMD, diabetic retinopathy (DR), CMV, vitreoretinopathy, and posterior uveitis [3]. Topical eye drops supply drugs to most of anterior segment tissues, whilst eye injections (most notably, intravitreal) are the standard drug administration option for posterior segment diseases. Poor bioavailability (less than 5%) represents a major issue with topically administered ocular medications, while invasiveness (typically repeated monthly intravitreal injections) and non-compliance issues are the main hurdles to treating the diseases of the posterior segment.

The barriers to treating diseases of the anterior segment include a tight corneal–epithelial junction, reflex blinking and tearing, ocular tissue/s metabolism, tear turnover, nasolacrimal drainage, efflux transporter pumps, and the blood–aqueous barrier [4]. These anatomical and physiological constraints have been discussed in detail elsewhere [3,5]. The main barrier to medication absorption following topical application is the corneal epithelium. Tight intercellular connections surround cells that are on the surface serve as barriers to prevent drug molecules from entering the cells through the paracellular route [6]. The typical drop size of topically instilled eye drops, which is delivered to the eye, is in the volume range of 25–56 µL. Although the human eye can temporarily accommodate up to 30 µL, any excess amount is quickly wasted due to reflex blinking, greatly reducing the amount of medication that is ultimately accessible for a therapeutic effect [7,8]. P-glycoprotein and multidrug resistant proteins primarily cause drug efflux. P-glycoprotein, which is located in the blood–aqueous and blood–retinal barriers [9,10], eliminates amphipathic substances, while multidrug resistant proteins, which are found in the ciliary body and blood–aqueous barrier [11], are known to export organic anions. Endothelial cells from the blood vessels in the iris and cilia form the blood–aqueous barrier together with the non-pigmented ciliary epithelium. This prevents the bulk of medications from reaching deeper ocular tissues and controls the diffusion of soluble molecules between the front and back of the eye by building tight connections at the cellular level [12,13].

For drugs targeted at the back of the eye, the retinal pigmented epithelium, ciliary body, and ocular metabolic enzymes reduce how much of the drug remains [13,14]. The posterior segment barriers include the inner limiting membrane, vitreous diffusion, tight retinal–pigmented epithelium junctions, and the blood–retinal barrier [15]. The inner limiting membrane is a substantial physical barrier that inhibits drugs from being delivered to the posterior portion of the eye [16]. The vitreous body represents a second major barrier for drug delivery for the posterior segment. In the human eye, the vitreous body, a transparent, gel-like substance, accounts for around 80% of the total volume. The vitreous body consists of extraordinarily high water content (>97%) and collagen fibers [17,18]. The collagen fibers make up the network that fabricate the gel structure in the 3D shape and make it flexible and strong against mechanical pressures. The vitreous body can act as a barrier either physiologically or anatomically. The physiological barrier action is represented by the slowing down of drug diffusion and the anatomical barrier is represented by the 3D gel-like structure [19]. The retina and retinal pigment epithelium’s limiting structure prevents the free flow or diffusion of therapeutic drugs, which is what gives the retina its tight junctions [20]. Another significant challenge to drug delivery to the posterior portion is the blood–retinal barrier. The outer and inner blood–retinal barriers make up the blood–retinal barrier. The inner blood–retinal barrier is made up of retinal capillary endothelial cells, whereas the outside blood–retinal barrier is composed of tightly connected retinal pigment epithelial cells. Similar to the blood–brain barrier, the absence of wide gaps in the retinal pigment epithelium and retinal endothelial cells prevents passive drug transport. Only very small molecules from the choroid, including carbon dioxide, oxygen, and lipophilic compounds, can diffuse to the inner retinal tissues [21].

In this review, the most common ocular chronic disorders will be discussed. These conditions necessitate longer treatment intervals with drugs, and the most effective drug delivery systems should ideally improve the activity, stability, and distribution of drug molecules to target the ocular tissues. The utilization of long-acting drug delivery systems (LADDS), particularly implantable drug delivery systems (IDDS), and their formulation and methods of characterization, assessment, and their clinical application are covered.

## 2. Chronic Eye Diseases That Require Long-Acting Therapy

### 2.1. Dry Eye Diseases

Dry eye diseases, which are characterized by symptoms such as ocular surface irritation and vision impairment, are brought about by insufficient tear production or tear hyperosmolarity [22]. Accordingly, dry eye diseases could be classified into deficient or evaporative diseases [23]. The tear film’s osmolality rises as a result, and the ocular surface becomes inflamed [24]. According to estimates, 5–30% of adults over 50 are at risk for developing dry eye diseases [25,26,27]. Increasing evidence suggests that ocular inflammation is a major contributor to the pathophysiology of dry eye because it has demonstrated that regardless of the origin of dry eye condition, proinflammatory cytokines and T helper cells are present on the ocular surface [28].

**Figure 1:**
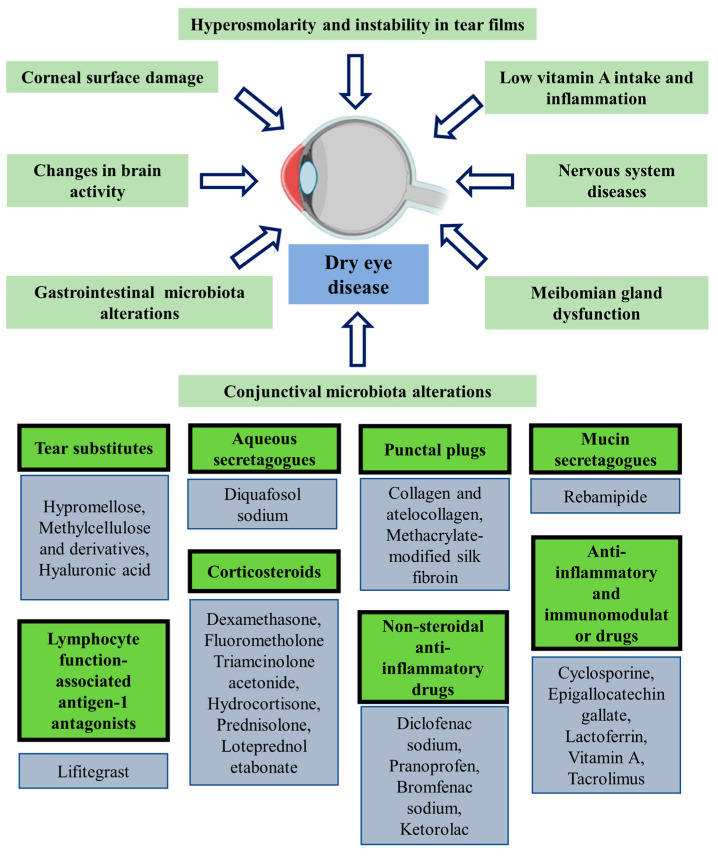
Major pathophysiological causes for the development of dry eye diseases.

There are various pathophysiological factors that might trigger dry eye diseases (Figure 1). The major etiological causes are ocular surface injury, meibomian gland dysfunction, and tear film hyperosmolarity and instability [29,30]. Thus, for dry eye to be defined with the greatest degree of precision and to be distinguished from other ocular surface disorders, the etiological factors are essential. In addition, the symptoms dry eye syndrome are associated with malfunction in particular brain regions [31]. In addition, gut microbiome disturbance or dysbiosis was identified to be associated with the development of dry eye, particularly primary Sjogren’s disease (Figure 1) [32].

To provide comfort to the ocular surface, tears are replenished with a variety of lubricants. These lubricants, which are called artificial tears, include several polymer solutions, such as hyaluronic acid, carboxymethyl cellulose, polyvinyl alcohol, polyethylene glycol, and polyvinyl pyrrolidone [33]. Products made of these polymers can be supplemented with additional additives to increase lubrication and prolong their duration in the eye because they do not include any physiologically bioactive molecules such as those found in real tears [34,35]. Diquafosol sodium and other aqueous secretagogues are useful for treating dry eye conditions and promoting mucin and tear production [36]. Punctal plugs, which are microscopic implants made of silicone or collagen, were initially developed to treat dry eyes by occluding the punctal duct, causing tear fluid to accumulate [37]. Topical glucocorticoid formulations have gained widespread acceptance as a temporary therapeutic option for dry eye diseases due to their well-recognized anti-inflammatory effects. Topical steroids have been demonstrated to have anti-inflammatory effects on a number of targets associated with the symptoms and signs of dry eye, such as lowering cytokine expression, maintaining the integrity of the corneal epithelium [38,39,40], and increasing tear production in animal models [41]. Topical glucocorticoid drops have been demonstrated to ameliorate symptoms and clinical indicators after a month of usage in various trials, and provided prominent lowering in the level of pro-inflammatory cytokines [40,41,42,43]. Nonsteroidal anti-inflammatory drugs were also employed topically to treat dry eye syndrome. Topical diclofenac and topical ketorolac have demonstrated enhanced effectiveness against dry eye syndrome [44,45]. Topical cyclosporine A, a typical immunomodulatory, reduces the number of T cells that are activated and the level of inflammatory markers in dry eye syndrome, as well; it controls inflammation, as well as the death of conjunctival epithelial cells [46,47,48]. Tacrolimus, which is a 10–100 times more potent immunosuppressant than cyclosporine A, is routinely used to treat dry eye diseases [49].

The continuous precorneal clearing caused by the dynamic nature of the ocular surface, together with blinking, nasolacrimal discharge and response, and basal tearing, all help to quickly remove foreign particles from the eye. Less than 20% of the applied dosage remains on the ocular surface after a single blink, providing a brief window for drug absorption (5–7 min) [50]. This is especially true when the quick turnover of tear fluid is taken into consideration. When two or more eye drops are applied at once, there is greater competition for space in the precorneal cavity, which can further reduce precorneal retention time and ocular bioavailability when treating dry eye diseases [51]. As a result, the development of better drug delivery systems might increase the efficacy of drugs used topically to treat dry eye disorders. Punctal plugs have been proven to increase the action of loaded medications in the treatment of dry eye diseases and to move beyond the ocular barriers [52,53,54]. The incorporation of mucin secretagogue rebamipide into nanocarriers significantly increased the activity and penetration into ocular tissues. The optimum use of cyclosporin A to treat dry eye disorders is hampered by its very hydrophobic nature and very poor water solubility [55,56]. Nanocarriers have been widely utilized to increase the activity, effectiveness, penetration, and duration of cyclosporin A [57]. The FDA had approved the use of several cyclosporine A drug delivery systems, such Restasis^®^, Ikervis^®^, and Cequa^®^, for the treatment of individuals with moderate-to-severe dry eye diseases [57,58,59]. Corticosteroids and non-steroidal anti-inflammatory drugs showed improved activity and bioavailability, reduced toxicity, and extended release upon incorporation into nanocarriers for ocular applications [60,61]. The drug delivery systems used to develop treatments for dry eye disease are summarized in Table 1.

### 2.2. Glaucoma

**Figure 2:**
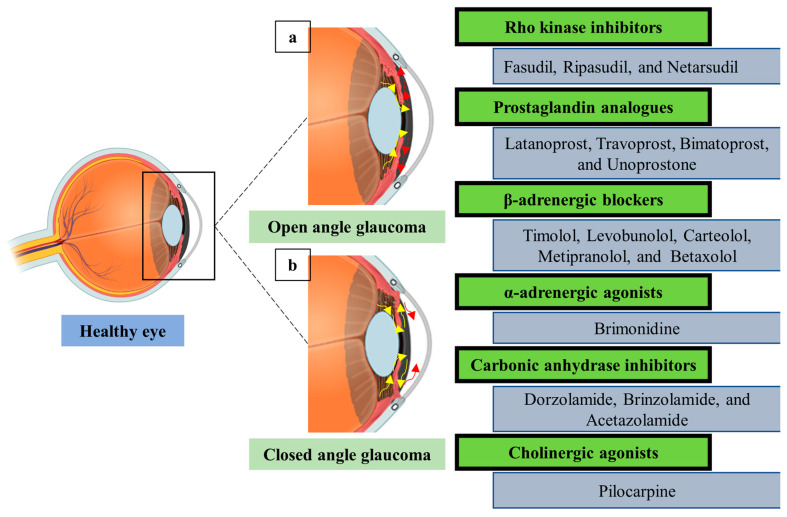
Schematic diagram for the aqueous humor drainage mechanisms in open-angle and angle-closure glaucoma. Intraocular pressure elevation may be a result of (**a**) open-angle or (**b**) angle-closure glaucoma. Open-angle glaucoma occurs as a result of pathological changes in the outflow tissues, which increase the resistance to aqueous humor outflow. Angle-closure glaucoma prevents aqueous humor outflow due to blocking of iridocorneal angle.

The progressive loss of retinal ganglion cells is a hallmark of the ocular neuropathy known as glaucoma [62,63]. Ganglion cell degeneration is currently untreatable, leading to a focus on slowing the disease’s development as the aim of glaucoma treatment [64]. Glaucoma is therefore seen as a chronic condition that needs ongoing management [65]. Glaucoma, a major contributor to irreversible blindness, affects over 80 million people globally today. More than 100 million people are anticipated to be afflicted by this blinding condition by the year 2040 [66,67]. The primary disease-related risk factor that can be modified is increased intraocular pressure [68]. Glaucoma can manifest in two different ways: open-angle and angle-closure. In open-angle glaucoma, the outflow channel is still accessible, but the outflow resistance is increased because of pathological alterations to the outflow tissue (Figure 2a). On the other hand, angle-closure glaucoma prevents the aqueous humor from leaving the anterior chamber of the eye because the iridocorneal angle is closed (Figure 2b).

The type of glaucoma that affects people most frequently is primary open-angle [69]. Consequently, common therapy choices include ocular drops with the goal of lowering the intraocular pressure, prostaglandin analogues, Rho-kinase inhibitors, β-adrenergic blockers, α-receptor agonists, carbonic anhydrase suppressors, and cholinergic agonists. They achieve this either by boosting the aqueous humor’s outflow through a unique method or reducing its formation [68,70].

The bioavailability of anti-glaucomatous medicines is only 1–7% inside the eye because of their short time on the corneal surface, poor corneal penetration, and quick drainage with the tear fluid [71,72]. Additionally, up to three applications each day limit the effectiveness of their therapeutic effects [72,73]. Therefore, the development of improved drug delivery systems may boost the effectiveness of medications utilized topically to successfully lower the intraocular pressure. Prostaglandin analogues, which are medications that are poorly soluble in water, are typically coupled with preservatives, such as benzalkonium chloride or polyquaternium, to make them more soluble. However, repeated instillation of these medications may irritate the ocular surface [74]. Thus, employment of nanocarriers into the formulation of prostaglandin analogues would reduce the dependence on the solubilization effect of the preservative and could lead to the development of preservative-free formulations [75]. Moreover, nanocarriers have been used effectively to enhance ocular bioavailability and therapeutic activity, as well as to reduce systemic toxicity of topically applied β blockers, α-adrenergic agonists, and carbonic anhydrase inhibitors [76,77,78]. A summary of the drug delivery systems used to enhance the therapeutic activity of anti-glaucomatous drugs is found in Table 1.

### 2.3. Uveitis

Uveitis is the inflammation of the uveal tract. The uveal tract, which is the middle part of the eye, is located between the retina on the inside and the sclera, conjunctiva, and anterior chamber on the outside and consists of the ciliary body, the choroid, and the iris [79]. Uveitis is considered the fourth most common reason for acquired blindness, especially for chronic uveitis, and is characterized by a high rate of related complications [80,81,82,83]. Uveitis is subcategorized according to the inflamed anatomical section into either the anterior, intermediate, or posterior, where the inflammation and accompanied leucocytes are present in the iris, vitreous humor, or choroid, respectively [79]. The concurrent presence of anterior, intermediate, and posterior uveitis is called panuveitis. Anterior uveitis, which is far more common than intermediate, posterior, or panuveitis, accounts for around 85% of all incidences of uveitis [84]. Corticosteroids, such as fluocinolone acetonide, difluprednate, fluormetholone, and triamcinolone acetonide, as well as immunomodulatory medications, including rapamycin, infliximab, and methotrexate, may be applied topically to treat anterior uveitis.

The inadequate bioavailability and the ocular tissues’ barrier properties prevent the transfer of administered medications to deeper ocular tissues, which may lead to the failure of the uveitis treatment. Consequently, the use of an efficient drug delivery system could enhance the bioavailability and improve the activity of ocularly applied corticosteroids and immunomodulatory medications [85,86]. As seen in Table 1, the use of different drug delivery systems led to the formation of more efficient treatment choices.

### 2.4. Endophthalmitis

The word “endophthalmitis” refers to an infection of the aqueous vitreous humors and/or the surrounding ocular tissues brought on by bacteria or fungi. Endophthalmitis is considered an uncommon eye disease; however, it may cause a severe type of inflammation and might result in irreparable vision loss. Endophthalmitis can be exogenous or endogenous depending on how the infection is transmitted to the eye. Exogenous endophthalmitis is most usually brought on by microorganisms that enter the eye through an infection in the cornea, surgery, or an eye injury. In contrast, endogenous endophthalmitis occurs when the bacteria or fungus enter the eye through the bloodstream [87]. Gram positive bacteria e.g., Staphylococcus aureus and Streptococcus species [88], as well as gram negative bacteria e.g., Klebsiella species and E. coli, are the major causes of endophthalmitis [88,89,90].

The current treatment for endophthalmitis involves repeated intravitreal injections of antimicrobial, antifungal, or antiviral agents. This procedure increases the complications and commonly results in blindness by causing irritation, ocular pain, a rise in the intraocular pressure, intraocular hemorrhage, a greater risk of retinal detachment, and retinal damage [91,92,93,94,95]. Consequently, the development of drug delivery systems for antimicrobial, antifungal, or antiviral agents could enhance ocular tissue penetration and activity noninvasively (Table 1).

### 2.5. Cytomegalovirus Retinitis

Cytomegalovirus (CMV) retinitis is still the most common ocular-invading virus in patients with acquired immunodeficiency syndrome (AIDS) [96,97]. Patients continue to be at risk for developing CMV retinitis predominantly as a result of either a delayed diagnosis of HIV infection or as a result of noncompliance, intolerance, or resistance to antiretroviral therapy [98]. Even though the prevalence of CMV retinitis has significantly decreased due to development of more effective treatments, CMV retinitis is still a major contributor to vision loss in AIDS patients managed with antiretroviral drugs [99]. Therefore, understanding the incidence rate and risk factors associated with the development of CMV retinitis is essential for both patients and medical professionals.

CMV retinitis could be controlled via intravitreal injection of antiviral drugs, such as ganciclovir, foscarnet, and cidofovir [100,101]. These drugs were produced in noninvasive sustained release nanocarrier formulations employing a range of drug delivery systems (Table 1).

### 2.6. Retinal Diseases

#### 2.6.1. Age-Related Macular Degeneration

**Figure 3:**
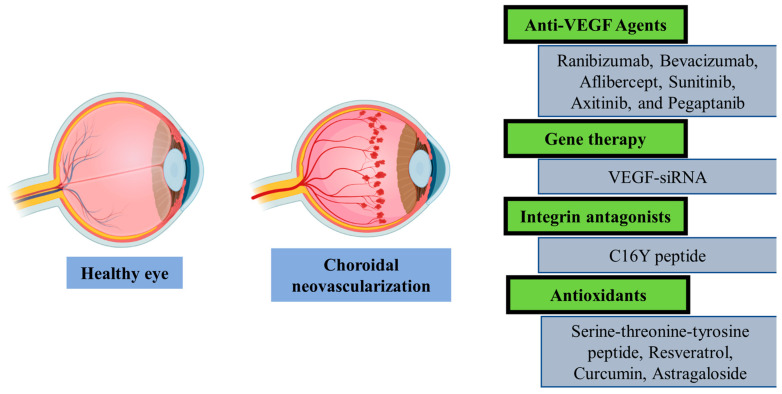
Schematic diagram for the process of choroidal neovascularization along with drugs, supplements, and biologics used for treatment of AMD.

Age-related macular degeneration (AMD) is a condition that damages the retina’s macular area and results in a gradual loss of the central vision clarity [102,103,104]. The incidence of AMD is rising progressively with age. The percentage of AMD cases in the United States increases from 2% at age of forty to about 25% by the age of eighty [105]. Wet and dry are two different types of AMD. A persistent disorder called dry AMD often causes some degree of visual impairment and sometimes leads to complete blindness. In contrast, wet AMD only affects about 15% of AMD patients; it manifests suddenly and, if untreated, advances quickly to blindness [106,107]. When AMD is first developing in the asymptomatic early stages, the retina develops drusen, which are aggregates of insoluble extracellular lipid and protein [108]. Although AMD typically never develops without antecedent drusen development, drusen regression is connected to the progression of intermediate AMD to geographic atrophy [109,110]. Geographic atrophy, which is one late stage of dry AMD, is characterized by dispersed zones of degeneration of the overlaying light-sensitive receptors of the retina, which depend on the retinal pigment epithelium cells for alimentary maintenance [111]. Choroidal neovascularization (Figure 3, CNV), another late stage of AMD in which newly immature blood vessels sprout from the choroid toward the retina, is considered a hallmark of the wet type of AMD [111,112]. Due to lack of rigidity, fluids leak around or into the retina from these blood vessels. The late stage of AMD includes the development of neovasculature. Thus, intraocular injections of medications that target vascular endothelial growth factor (VEGF), one of the key molecules in the development of neovascularization, have been shown to be particularly effective [113,114]. Tyrosine kinase inhibitors are furthermore utilized in AMD to reduce choroidal neovascularization [115]. VEGF can activate CNV via binding to two receptors, VEGFR-1/flt-1 and VEGFR-2/KDR, both of which have intrinsic tyrosine kinase activity. Small molecule tyrosine kinase inhibitors are used to disrupt this pathway [116]. Patients with early, moderate, or atrophic AMD, however, are not eligible for any form of treatment. Additionally, there are no effective ways to stop the transition from early to advanced phases at this time [117,118].

Anti-VEGF agents are not readily able to cross the biological membranes that limit their therapeutic activity in the management of AMD [119]. Modern drug delivery systems could make the currently prescribed treatments more effective and delay this change (Table 1).

#### 2.6.2. Diabetic Retinopathy

**Figure 4:**
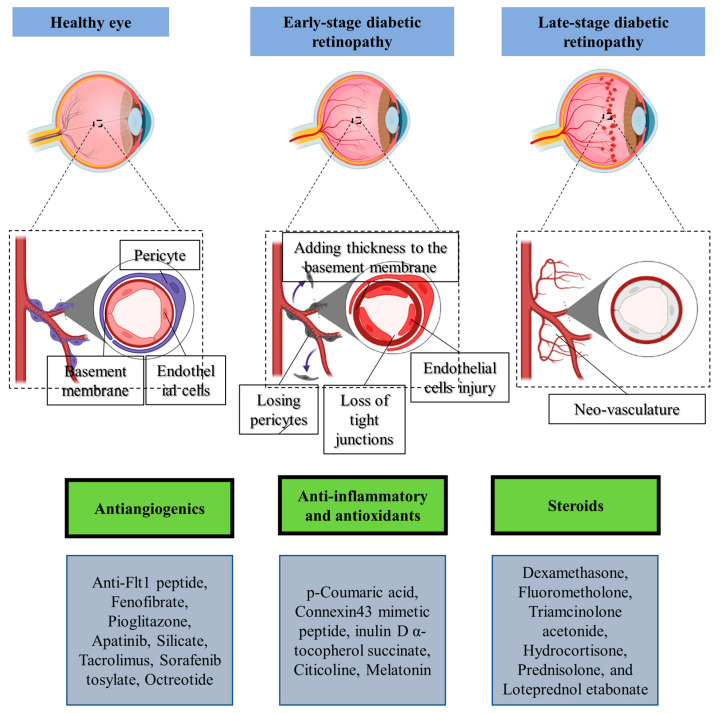
Schematic diagram for the progressive stages for diabetic retinopathy showing the early and late stages, along with drugs, supplements, and biologics used for treating diabetic retinopathy.

Diabetic retinopathy, which is a microvasculature diabetes-related problem, continues to be a predominant cause of vision loss and preventable blindness in individuals aged 20 to 74, especially in middle- and high-income nations [120]. Globally, an estimated 415 million people had diabetes in 2015, and by 2040, that figure is projected to increase to 642 million [121]. The number of those who have diabetic retinopathy and visual impairment is growing globally due to the rising incidence of diabetes and the increasing number of diabetics living longer [122]. The primary pathophysiology of diabetic retinopathy is a combination of changes brought on by hyperglycemia that leads to neovascularization (Figure 4). The neovascularization is caused by increased retinal vascular permeability, increased thickness of the retinal capillary basement membrane, inadequate blood supply to the tissues, and the release of numerous vasoactive molecules. The neovasculature is usually fragile, unstable, and leaky, causing retinal detachment and vitreous bleeding. Diabetic retinopathy combined with neovasculature is usually referred to as proliferative diabetic retinopathy, which can ultimately cause vision loss. Contrarily, the subtype of diabetic retinopathy known as non-proliferative diabetic retinopathy lacks neovascularization in the early stages. The development of microaneurysms and the minor dilatation of retinal blood vessels, which are recognized as early clinical indications of diabetic retinopathy, are common features of non-proliferative diabetic retinopathy [123,124]. The most common cause of visual loss in those with diabetic retinopathy is diabetic macular edema. In diabetic macular edema, the macula swells or thickens as a result of fluid building up sub- and intra-retinally and inside the macula as a result of the collapse of the blood–retinal barrier [125].

Antiangiogenics, steroids, anti-inflammatories, and antioxidant medications are the most popular treatments for diabetic retinopathy. However, most of these medicaments have poor ocular penetration and require implantation via surgery. Therefore, the development of more advanced drug delivery technologies may improve the potency of currently prescribed drugs, as shown in Table 1.

**Table 1:** Chronic eye conditions, available therapies, and drug delivery systems and their merits.

Disease	Treatment		Drug	Delivery System Platform	Advantages of Delivery Systems In Vivo	Refs.
Dry eye syndrome	Tear substitutes		Hypromellose	Solution		[126]
			Methylcellulose and derivatives	Solution		[127]
			hyaluronic acid	Solution		[128]
	Aqueous secretagogues		Diquafosol sodium	Solution		[129]
	Punctal plugs		Collagen and atelocollagen	In situ hydrogel	Prolonged activity	[49,50,130]
			methacrylate-modified silk fibroin	In situ hydrogel	Prolonged activity	[54]
	Mucin secretagogues		Rebamipide	Nanoparticles	Sustained release	[131]
				Liposomes	Improved activity	[132]
				Micelles	Improved penetration	[133]
	Anti-inflammatory and immunomodulatory drugs		Cyclosporine	Micelles	Improved activity	[134]
				Self-nanoemulsifying	Improved efficacy	[135]
				Liposomes	Improved activity	[136]
				Nanoparticles	Improved activity	[137]
				Nano-emulsion	Improved penetration	[138]
				Solid lipid nanoparticles	Controlled release	[139]
				In situ hydrogel	Improved activity	[134]
			Epigallocatechin gallate	Nanoparticles	Extended activity	[140]
				In situ gels	Enhanced efficacy	[141]
			Lactoferrin	Nanoparticles	Enhanced efficacy	[142]
				Nanocapsules	Controlled release	[143]
				Liposomes	Reduced irritation	[144]
				Nanostructured lipid carriers	Controlled release	[145]
			Vitamin A	Liposomes	Improved activity	[146]
			Tacrolimus	Nanoparticles	Improved penetration	[147]
				Progylcosomes	Improved activity	[148]
				Microcrystals	Improved efficacy	[149]
				Liposomes	Improved retention time	[150]
				Micelles	Prolonged activity	[151]
				Nanocapsules	Improved activity	[152]
	Corticosteroids		Dexamethasone	Dendrimer	Improved activity	[153]
				Nano-wafer	Improved activity	[154]
				Nanostructured lipid carriers	Improved activity	[155]
				Nanoparticles	Improved penetration	[156]
				Micelles	Release modulation	[157]
				Nanosuspension	Prolonged activity	[158]
				Nano emulsion	Improved activity	[159]
				Nanosponges	Improved permeability	[160]
			Fluorometholone	Nanoparticles	Improved activity	[161]
			Triamcinolone acetonide	Micelles	Release modulation	[60]
				Nanoparticles	Improved activity	[162]
			Hydrocortisone	Nanosuspension	Prolonged activity	[158]
				Micelles	Improved targeting	[163]
				Nanoparticles	Improved penetration	[163]
				Nanosuspension	Prolonged activity	[158]
			Prednisolone	Nanoparticles	Prolonged activity	[164]
				Nano capsules	Reduced toxicity	[165]
			Lotep rednol etabonate	Nanoparticles	Improved penetration	[166]
	Non-steroidal anti-inflammatory drugs		Diclofenac sodium	Nanoparticles	Improved bioavailability	[167]
				Nanosuspension	Prolonged activity	[168]
			Pranoprofen	Nanosuspension	Improved activity	[169]
				Nanoparticles	Improved activity	[169,170]
			Bromfenac sodium	Liposomes	Extended release	[171]
				Nanoparticles	Improved permeation	[172]
				Cubosomes	Improved bioavailability	[61]
			Ketorolac	Nanoparticles	Improved delivery	[173]
	lymphocyte function-associated antigen-1 antagonists		Lifitegrast	Solution		[174,175]
Glaucoma	Prostaglandin analogues		Latanoprost	Nanoparticles	Controlled release	[176]
				PEGylated solid lipid	Improved permeability	[177]
				Micelles	Extended release	[178]
				Cubosomes	Sustained release	[179]
				Nanoparticles	Improved permeability	[180]
			Travoprost	Gold nanoparticles	Improved stability	[181]
				Liposomes	Sustained release	[182]
				Spanlastics	Prolonged activity	[183]
				Nanoemulsion	Improved pharmacokinetics	[75]
				Implant	Controlled release	[184]
			Bimatoprost	Nanoparticles	Improved therapeutic activity	[185]
				Gold nanoparticles	Controlled release	[186]
				Nanoparticle hydrogel	Controlled release	[187]
				Microemulsion	Improved permeability	[188]
				Graphene oxide-laden	Controlled release	[189]
				Implants	Sustained release	[190]
				Nanovesicular systems	Sustained release	[191]
				Inserts	Extended release	[192]
			Unoprostone	Transscleral device	Sustained release	[193]
	Rho kinase inhibitors		Fasudil	Liposomes	Enhanced bioavailability	[194]
				Microspheres	Sustained release	[195]
			Ripasudil	Solution		[196]
			Netarsudil	Solution		[197]
	β-adrenergic blockers		Timolol	Nanoparticles	Extended release	[198]
				Micelles	Extended release	[178]
				Cubosomes	Improved bioavailability	[199]
				Nanogel	Sustained release	[200]
				Gelatinized core liposomes	Improved encapsulation	[201]
				Microemulsion	Improved bioavailability	[202]
			Levobunolol	Nanoparticles	Extended release	[203]
				Microparticles	Sustained release	[76]
			Carteolol	Nanocapsules	Improved activity	[204]
				Nanoparticles	Improved activity	[205]
				Chitosomes	Improved penetration	[206]
			Metipranolol	Nanocapsules	Reduced systemic side effects	[207]
			Betaxolol	Liposomes	Extended activity	[208]
				Nanoparticles	Controlled release	[209]
				Niosomes	Improved bioavailability	[210]
				Bilosomes	Improved transcorneal permeation	[211]
	α-adrenergic agonists		Brimonidine	Nanoparticles	Sustained release	[212]
				Inserts	Controlled release	[213]
				Niosomes	Sustained release	[214]
				Microspheres	Sustained release	[215]
				Liposomes	Improved effectiveness	[216]
				Implant	Sustained release	[217]
				Gelatin-core liposomes	Improved drug loading	[77]
	Carbonic anhydrase inhibitors		Dorzolamide	Nanoparticles	Improved activity	[218]
				Nanoemulsion	Enhanced ocular delivery	[219]
				Liposomes	Prolonged action	[78]
				Microparticles	Sustained release	[220]
				Niosomes	Improved activity	[221]
				Implant	Extended drug delivery	[222]
				Inserts	Improved activity	[223]
			Brinzolamide	Nanoparticles	Improved therapeutic activity	[224]
				Nanocrystals	Improved penetration	[225]
				Liposomes	Sustained release	[226]
				Nanocapsules	Improved bioavailability	[227]
				Nanoemulsion	Improved therapeutic efficacy	[228]
				Nanofibers	Improved patient compliance	[229]
				Implant	Sustained release	[230]
			Acetazolamide	Cubosomes	Improved therapeutic efficacy	[231]
				Spanlastics	Enhanced ocular delivery	[232]
				Transgelosomes	Enhanced ocular delivery	[233]
				Implants	Sustained release	[234]
				Niosomes	Improved permeability	[235]
				Bilosomes	Improved permeability	[236]
				Microsponges	Improved therapeutic efficacy	[237]
				Dendrimers	Sustained release	[238]
	Cholinergic agonists		Pilocarpine	Nanoparticles	Sustained release	[239]
				Nanocapsules	Improved bioavailability	[240]
				Dendrimers	Prolonged residence time	[241]
Uveitis	Corticosteroids		Fluocinolone acetonide	Implant (Retisert^®^)	Sustained release	[242]
				Nanoparticles	Improved bioavailability	[243]
			Difluprednate	Microneedles	Sustained release	[244]
			Fluormetholone	Nanoparticles	Improved penetration	[245]
				Nanocrystals	Improved sustained activity	[246]
			Triamcinolone acetonide	Nano lipid carriers	Improved penetration	[247]
	Immunomodulator drugs		Adalimumab	Hydrogel	Improved permeability	[248]
			Infliximab	Liposomes	Prolonged activity	[249]
			Methotrexate	Implant	Sustained release	[250]
			Sirolimus (Rapamycin)	Implant	Extended release	[251]
				Micelles	Sustained release	[252]
				Exosomes	Improved therapeutic activity	[253]
				Liposomes	Improved therapeutic activity	[86]
Endophthalmitis	Antimicrobials		Daptomycin	Nanoparticles	Noninvasive and improved activity	[254]
			Vancomycin	Nanostructured lipid carriers	Improved permeability and activity	[255]
				Nanoparticles	Sustained release	[256]
				Thermoresponsive hydrogels	Controlled release	[257]
				Liposomes	Improved permeability	[258]
				Implant	Controlled release	[259]
				Niosomes	Improved permeability	[260]
			Ceftazidime	Nanoparticles	Improved activity and permeability	[261]
	Antifungals		Amphotericin B	Liposomes	Improved activity-reduce toxicity	[262]
			Voriconazole	Thermo-sensitive in situ gel	Sustained release	[263]
				Nanoparticles	Improved permeability	[264]
				Microemulsion	Controlled release	[265]
				Elastosomes	Improved activity and reduced toxicity	[266]
				Micelles	Improved stability	[266]
				Liposomes	Improved permeability	[267]
	Antivirals		Cidofovir	Micelles	Prolonged activity	[268]
				Liposomes	Prolonged activity	[269]
			Foscarnet	Liposomes	Improved activity and permeability	[270]
			Ganciclovir	Nanoparticles	Sustained release	[271]
				Glycerosomes	Sustained release	[272]
				Microemulsion	Improved permeability	[273]
				Vitrasert	Prolonged activity	[274]
				Minitablets	Sustained release	[275]
Retinal diseases	Age-related macular degeneration	Anti-VEGF Agents	Ranibizumab	Nanoparticles	Improved activity	[276]
			(Antibody fragment)	Microparticles	Improved intravitreal delivery	[277]
				Liposomes	Increased encapsulation-release	[278]
				Quantum dots	Sustained release	[279]
				Implant	Sustained release	[280]
			Bevacizumab	Nanoparticles	Sustained delivery	[281]
			(Monoclonal antibody)	Bi-layered capsule	Sustained delivery	[282]
				Nanocapsules	Improved bioavailability	[283]
				Implant	Sustained release	[284]
				Microparticles	Sustained release	[285]
				Liposomes	Sustained release	[286]
			Aflibercept (VEGF-Trap)	Nanoparticles	Sustained drug release	[287]
				Microspheres	Extended release	[288]
			Sunitinib	Nanoparticles	Superior prolonged activity	[289]
				Micelles	Extended release	[290]
			Axitinib	Nanoparticles	Superior activity	[291]
			Pegaptanib	PEGylated aptamer	Prolonged activity	[113]
		Gene therapy	VEGF-siRNA	Liposomes	Improved activity-stability	[292]
				Nanoball	Improved activity-targeting	[293]
				Nanoparticles	Improved therapeutic activity	[294]
		Integrin antagonists	C16Y peptide	Nanoparticles	Sustained release	[295]
		Antioxidants	Serine-threonine-tyrosine peptide	Nanoparticles	Targeting	[296]
			Resveratrol	Nanoparticles	Sustained release	[297]
			Curcumin	Liposomes	Improved activity	[298]
			Astragaloside	Nanocapsules	Improved activity	[299]
	Diabetic retinopathy	Antiangiogenics	Anti-Flt1 peptide	Nanoparticles	Sustained release	[300]
				Micropump implant	On-demand targeting	[301]
			Fenofibrate	Nanoparticles	Controlled release	[302]
			Pioglitazone	Nanoparticles	Controlled/improved activity	[303]
			Apatinib	Nanoparticles	Improved activity	[304]
			Silicate	Nanoparticles	Improved activity	[305]
			Tacrolimus	Nanoparticles	Improved activity	[306]
			Sorafenib tosylate	Nanoparticles	Improved activity	[307]
			Octreotide	Nanoparticles	Improved activity-targeting	[308]
		Anti-inflammatory and antioxidants	p-Coumaric acid	Nanoparticles	Improved activity	[309]
			Connexin43 mimetic peptide	Nanoparticles	Targeting	[310]
			Inulin D α-tocopherol succinate	Nanomicelles	Improved activity	[311]
			Citicoline	Liposomes	Improved permeation	[312]
			Melatonin	Nanoparticles	Controlled release and enhanced tolerability	[312]

## 3. Overview of Ocular Delivery Systems

**Figure 5:**
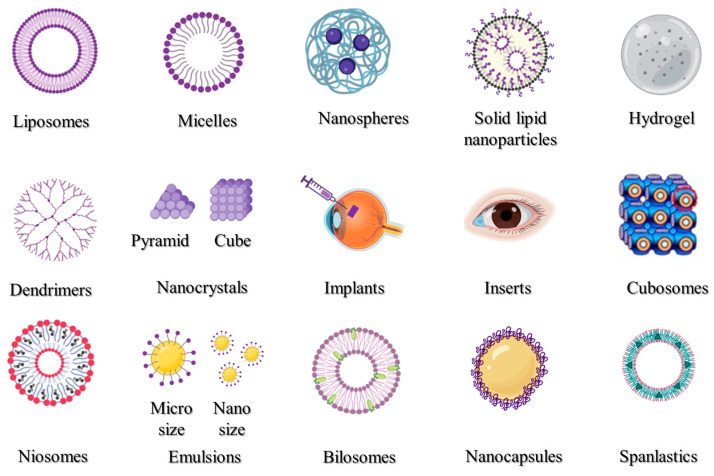
Nanocarrier systems investigated for ophthalmic uses.

Many disorders of the anterior region of the eye may be efficiently treated via topical administration; however, it is more challenging to target conventional therapeutic doses to the posterior of the eye in this manner. Thus, various nanocarriers have been created and investigated for the transport of drugs and genes to the anterior or the posterior portions of the eyes. The most popular nano-drug delivery systems are depicted in Figure 5, and these can be utilized to increase the activity and bioavailability, and/or lessen the toxicity of the active pharmaceutical ingredients used. Liposomes, nanoparticles, micelles, inserts, implants, hydrogel, and emulsions are some of the most frequently utilized drug delivery systems.

### 3.1. Liposomes

Liposomes (Figure 5) are closed vesicles made of a phospholipid bilayer that can contain both drugs that are soluble in fat [313] and those that are soluble in water [314]. Due to their biodegradability, biocompatibility, and capacity to serve as drug carriers, liposomes have been thoroughly investigated for topical ocular administration (Table 1). Liposomes are particularly useful for large molecular weight and inadequately water-soluble drugs because they promote drug permeation through ocular tissues by virtue of their superior spreading ability and rheological properties that enable prolonging the drug availability on the surface of the eye [86,315]. Liposomes’ amphiphilic lipids form a tear compound-interacting sublayer when they make contact with tear lipid components. Polar heads and tails face the polar and non-polar tear lipid components, respectively, and help distribute the medication throughout the ocular surface [316]. Extensive research has examined the merits of liposomes for ocular use, minimizing potential drug toxicity and improving their absorption and bioavailability compared to an unencapsulated drug. These medications include vancomycin, tobramycin, ganciclovir, fluconazole, brinzolamide, triamcinolone acetonide, and cyclosporine A. Drug-loaded liposomal formulations injected intravitreally have a number of benefits. Some benefits include lengthening the half-life of drugs [317], safeguarding labile compounds [318], and prolonging the time that liposomes spend in the tissues of the eye [319].

### 3.2. Polymeric Micelles

Amphiphilic polymers can self-assemble into different structures, known as micelles (Figure 5). The formation of micelles within the nanometer range can efficiently improve the aqueous stability and enhance cell permeability. Prior research has demonstrated that the use of a nanomicelle formulation increased medication absorption in the eye [320,321]. Nanomicelle formulations are primarily used to improve the solubility of medications with low solubility and subsequently improve their bioavailability. Hesperidin, Sirolimus, Voriconazole, and Sunitinib are only a few of the medications that were made into polymeric nanomicelles with better solubility and therapeutic efficacy (Table 1).

### 3.3. Polymeric Nanoparticles

Polymeric nanoparticles (Figure 5) could be produced by the use of naturally occurring or synthetically produced polymers. Chitosan, hyaluronic acid, carboxymethylcellulose sodium, albumin, and sodium alginate are some examples of natural polymers, whereas poly(lactic-co-glycolic acid), poly(-caprolactone), and poly(ethylene glycol) are examples of synthetic polymers [322]. Some retinal drugs are currently not performing as expected due to the physical and chemical properties of the medications, as well as the distinctive anatomical structure of the eye. The bioavailability of these drugs was significantly increased [323], their toxicity was reduced [324], invasive procedures could be avoided [325], and pharmacokinetic modulation was achieved [326] via incorporation into polymeric nanoparticles (Table 1). These drugs include dexamethasone, cyclosporin, latanoprost, voriconazole, and ganciclovir. Hyaluronic acid, polyethylene glycol, and chitosan are examples of mucoadhesive polymers that may be employed to alter nanoparticles to lengthen their pericorneal residence duration [327]. Moreover, mucus penetrating nanoparticles, which possess low surface tension, low viscosity, and higher hydration water content, can enhance the penetration of therapeutic medicines through the cornea, increasing their bioavailability and resulting in better pharmacologic results. Consequently, mucus penetrating nanoparticles may significantly improve the treatment of posterior ocular problems, which include posterior uveitis, CMV, and retinal disorders [328].

### 3.4. Solid Lipid Nanoparticles

Lipids have been used to ameliorate the limited water solubility of several lipophilic drugs and adapt them as a drug delivery system [329]. Müller and Lucks initially developed solid lipid nanoparticles (SLNs, Figure 5) in 1996, which received the attention of scientists as a popular, stable, safe, and effective nanoscale drug delivery device. A surfactant layer that surrounds a solid lipid core in SLNs stabilizes and holds the medication [330]. Drug molecules can be found mostly in the center of particles or molecularly scattered throughout the matrix, depending on the drug solubility and the drug/lipid ratio [331]. SLNs are considered an efficient system intended for ocular drug delivery. SLNs can improve corneal drug absorptivity, enhance ocular tissue penetration and bioavailability, prolong residence time, and provide extended drug release properties [332]. SLNs were efficiently used to improve the delivery of bimatoprost [185], ofloxacin [333], and dorzolamide [334], as shown in Table 1.

### 3.5. Hydrogels

Hydrogels (Figure 5) are produced when polymeric solutions are crosslinked to form a network. The hydrogel complexation is formed on the basis of hydrophilic interactions between the polymer tail and water molecules [335]. Hydrogels are widely employed to provide ocularly applied or injectable dosage forms to a variety of eye regions. For ocular application, there are various hydrogel formulations that have FDA approval. A hydrogel sealant called ReSure^®^ has been authorized for use in the non-operative treatment of clear corneal incisions. Hydrogels were also used to formulate and enhance the therapeutic activity of ocularly applied drugs, such as dexamethasone [336], bevacizumab [337], and timolol [338], as shown in Table 1.

### 3.6. Dendrimers

Dendrimers (Figure 5) are globular, negatively, positively, or neutrally branch-like nanostructured polymers. They derive their net charge from the functional groups, which are located at the ends of their branches [339]. These molecules consist of a fundamental unit called the “core”, which comprises the major component, and side chain units called “dendrons” [340]. Drugs may be conjugated to the ligands on the dendrimer surface or may be retained in the dendrimer core. Dendrimer manufacturing, generation, surface characteristics, and conjugation technique all have an impact on the drug-loading and drug-release kinetics of dendrimers [341]. As a result of their ability to selectively target inflammatory cells while causing no harm to healthy tissue, dendrimers have proven to be a viable drug delivery vehicle for the treatment of inflammatory eye conditions. The capacity to lower medication toxicity off-target is the key advantage of dendrimers’ targeting abilities [153]. Utilizing dendrimers effectively can increase the therapeutic effectiveness of various active pharmaceutical compounds (Table 1), including pilocarpine [241], tropicamide [241], dexamethasone [342], brimonidine, and timolol [343].

### 3.7. Nanocrystals

Nanocrystals (Figure 5) are crystals of therapeutic drugs with particle sizes as small as a few hundred nanometers, where pure drug crystals may occasionally be stabilized by the addition of surface active agents or polymeric solutions [344]. The benefits of nanocrystals over conventional nanocarriers, such as their high drug payload and comparative ease of manufacture, make them appealing candidates for the delivery of medications that are not readily water soluble [345,346]. The preparation of therapeutic drugs in the form of nanocrystals for ocular administration has various advantages. These advantages include better tolerability, increased ocular absorption, providing intermediated and prolonged release of drugs in the eye, and improved ocular permeation [347]. They also include improved ocular safety, increased formulation retention in cul-de-sac, and enhanced ocular permeation [152]. A number of medications used ocularly have been transformed into nanocrystals (Table 1) with enhanced properties, and these include dexamethasone [348], itraconazole [349], tedizolid [350], and brinzolamide [227]. Moreover, Novartis Pharmaceutical Corporation’s formulation of nepafenac nanocrystals received approval for commercial release (FDA, 2012) under the brand name Ilevro^®^.

### 3.8. Cubosomes

Cubosomes (Figure 5) are made up of two inner aqueous pathways that are separated into two arched interpenetrating lipid bilayers, which are structured in three dimensions resembling honeycombs [351]. These pathways can be occupied by a variety of bioactive molecules, including natural bioactives, chemical pharmaceuticals, peptides, polypeptides, and proteins [352]. Cubosomes are thought to be promising delivery systems because of their special characteristics, including thermodynamic stability, bioadhesion, the capacity to encapsulate different types of drugs, and their potential to control drug release [353]. Active medicines and macromolecules can successfully be applied topically to the posterior portion of the eye using cubosomes (Table 1). These drugs include beclomethasone [352], flurbiprofen [354], timolol [199], and brimonidine [355].

### 3.9. Niosomes

Niosomes, which are a type of vesicular system that includes a non-ionic surfactant, are closed bilayer structures produced once the nonionic surfactants self-assemble in an aqueous media to create nanocarriers (Figure 5). Researchers have begun using niosomal systems to treat severe inflammatory diseases and conditions, such various malignancies, because of their potential to boost the bioavailability and efficiency of the encapsulated therapeutics [356]. Niosomes are being investigated more and more for improving drug delivery to both segments of the eye, anterior and posterior, as well as promoting drug penetration and retention in ocular tissues. As a consequence, niosomes showed a considerable increase in the absorption and transcorneal permeability of topically applied drugs at the ocular surface (Table 1). These drugs include cyclopentolate [357], voriconazole [358], acetazolamide [359], gentamicin [360], brinzolamide [361], pilocarpine [362], and tacrolimus [363]. Additionally, niosomes, particularly charged vesicles, have been effectively used to transfer genes by subretinal or intravitreal injection to the retinal area [364].

### 3.10. Emulsions

An emulsion (Figure 5) is a uniform dispersion system that is formed upon mixing two or more immiscible liquids under certain circumstances [365]. Lipid-based emulsions have become a potential vehicle for ocular medication administration. The emulsions enhance ocular delivery using one of two major strategies, either by improving ocular permeability or by lengthening the period the formulation is retained on the ocular surface [366]. Both hydrophilic and lipophilic drug types may be loaded into emulsions [367,368]. Emulsions have been successfully used to create more effective formulations for several medications used intraocularly that have increased absorption and therapeutic effectiveness. These drugs include cyclosporine A [369], coumarin-6 [370], azithromycin, and disulfiram [371].

### 3.11. Bilosomes

One type of vesicular drug delivery system is the bilosome (Figure 5), which is made up of non-ionic amphiphilic compounds with integrated bile salt molecules. The negatively charged bile salts serve to maintain the bilosomal structure [372]. In comparison to niosomes and liposomes, these drug carriers are more stable and can effectively increase drug absorption through biological membranes [373]. Moreover, bilosomes can enhance the permeability of polysaccharides, proteins, and polypeptides, which are poorly transported through mucosal epithelial cells [374]. Previous research studies have assessed the effectiveness of bilosomes in the administration of ocular drugs (Table 1) and found that bilosomes are well tolerated by corneal tissues [236]. These drugs include terconazole [375], acetazolamide [236], ciprofloxacin [376], ciprofloxacin [376], agomelatine [377], and betaxolol [211].

### 3.12. Nanocapsules

Nanocapsules (Figure 5) are a subtype of nanoparticles that are comparable to vesicular systems, in which a medicine is contained in a hollow vessel with an inner liquid core encircled by a polymeric coating [378]. Nanocapsules are well-known to be retained in the cornea for a prolonged time and to enhance penetration throughout the deep ocular tissues [152]. Thus, the development of topically applied drug-loaded nanocapsules could reduce uncomfortable intravitreal injections and systemic delivery, which have serious side effects [152]. The therapeutic action of several medications was effectively potentiated via formulation in the form of nanocapsules (Table 1). These drugs include bevacizumab [379], prednisolone [165], tacrolimus [152], brinzolamide [227], and cyclosporine [380].

### 3.13. Spanlastics

Elastic niosomes, also known as spanlastics (Figure 5), are a subtype of vesicular drug delivery systems that are relatively new to the market. They resemble niosomes (non-ionic surfactant vesicles), except they contain an edge activator. They were first described as systems for ocular drug delivery [381], but since then, they have been used to deliver medications to a variety of bodily organs. The spanlastics’ bilayers become more elastic and deformable when an edge activator is present, which improves drug absorption across biological membranes. Spanlastics were efficiently used to payload hydrophilic, hydrophobic, and amphiphilic therapeutic pharmaceuticals for ocular use, especially the delivery to the posterior segment (Table 1). These drugs include ketoconazole [381], cyclosporine A [382], clotrimazole [383], and vanillic acid [384].

## 4. Long-Acting Ocular Drug Delivery Devices

### 4.1. Solid Devices

Solid ocular devices are applied to the eye in a solid form and include inserts, implants, contact lenses, and films. Ocular inserts are objects that could be loaded with therapeutic drugs and inserted into the conjunctival sac for extending the duration of medicine delivery. Based on their physicochemical characteristics, the inserts are divided into three categories: bioerodible, soluble, and insoluble [385]. Soluble and erodible devices gradually dissolve while dispensing the medication and require no need for removal, while insoluble inserts can typically distribute medications at a controlled, predetermined rate through reservoir and matrix systems, but they must be removed from the eye [386]. The system prolongs drug activity, increases drug residency, improves bioavailability, and prevents crest and trough release profiles to subvert the negative effects that go along with those features [385]. Bimatoprost [387], acyclovir [388], triamcinolone acetonide [389], voriconazole [390], ketorolac [391], azithromycin [392], and dorzolamide [223] are just a few of the medications that have been delivered non-invasively to the eye using ocular inserts. A list of the commercially available long-acting drug delivery systems is shown in Table 2.

Ocular implants are solid devices that are used as medication delivery systems to slowly release molecules from polymeric matrices that are either biodegradable or not over the course of months to years. Contrary to non-biodegradable implants, which must be surgically removed after treatment, biodegradable solid implants are made utilizing biodegradable polymers, including polycaprolactones, polyglycolic acid, polylactic acid, and polylactic-co-glycolic acid, and polyanhydrides. However, these implants can have unpredictable drug release characteristics [431]. These implants can be placed at different sites in the eye, including the cameral, vitreal, scleral, episcleral, and subconjunctival areas. Implants have a number of benefits over more conventional means of administering medication to the eye, including bypassing the blood–ocular barrier and delivering a defined drug amount directly to the target site for a long period. Therefore, the danger of infection or retinal detachment may be reduced with the use of implants placed intravitreally, which may also localize therapy to the vitreous with minimal exposure to the systemic circulation [412]. In addition, implants minimize the need for repeated treatments by continuously supplying medication over a long period and are consequently suitable as a treatment of long-term eye disorders. Durasert^TM^ is a solid polymer implant technology in which small drug molecules can be released for up to three years. Three FDA approved implants using this technology including Iluvien^®^, Retisert^®^, and Vitrasert^®^ [432].

Drug-eluting contact lenses are solid dosage forms that have high potential to produce prolonged drug residence and close drug contact with the cornea, resulting in a major enhancement of drug bioavailability [433]. Consequently, drug-laden contact lenses provide several advantages, such as lowering the overall amount of medication required, decreasing dosing frequency, and diminishing the quantity of medication lost through systemic absorption [434]. Molecular imprinting, supercritical fluids, ion ligation, and colloidal polymeric nanoparticles are a few techniques that have been designed to payload pharmaceuticals into contact lenses [435]. Many medications have been placed into contact lenses in an effort to increase their pharmacological activity and move past the eye’s barriers, which hinder drug delivery, especially for the posterior chamber of the eye. These medications include dexamethasone [436], phomopsidione [437], latanoprost [438], a timolol–bimatoprost combination [433], and flurbiprofen [439]. Before some of these technologies can be used in clinical settings and made commercially available, a number of problems need to be resolved, including protein adhesion, diversity in the swelling ability, changes in water content, opacity, surface integrity, strength properties, ion and oxygen permeation, and drug leakage during manufacturing and storage.

Ocular films are solid sterile dosage forms that are applied topically on the eye sac to improve ocular bioavailability and remove barriers to ocular drug delivery [440]. The use of ocular films improves therapeutic efficacy, reduces systemic adverse effects, and minimizes dose frequency. In order to maximize the therapeutic response and patient compliance, ocular films could present intriguing prospects as a vehicle for the administration of therapies. They could thus replace the conventional dosage forms. However, the designation of efficient films for the ocular delivery of therapeutic medications depends on a thorough understanding of the medication, the restrictions of drug permeation to ocular tissues, and the excipients employed. The construction of ocular films should therefore take into account each of these elements. In an effort to maximize their therapeutic action, a plethora of medications were administered as ocular films, including acetazolamide [441], timolol maleate [440], ofloxacin [442], fluconazole [442], and dorzolamide hydrochloride [443].

### 4.2. Microneedles

**Figure 6:**
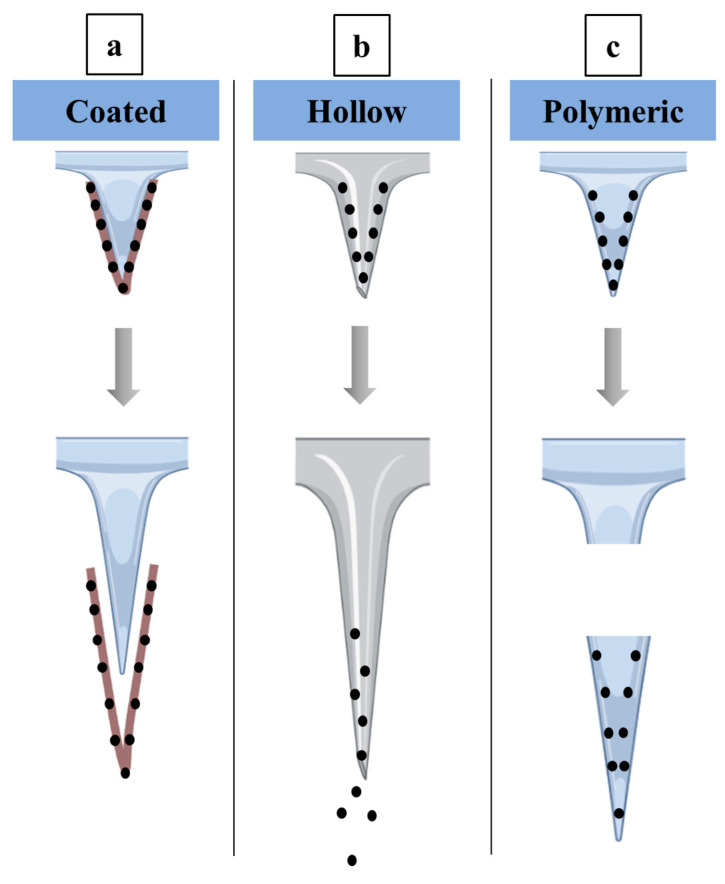
Different types of microneedles frequently used for ocular drug delivery. Ocularly applied microneedles can be classified into (**a**) solid-coated, (**b**) hollow, and (**c**) polymeric microneedles. (**a**) Solid coated microneedles are used to puncture the ocular tissue and allow the coated medication to disperse. (**b**) Hollow microneedles are tiny needles that completely contain the medication formulation. (**c**) Polymeric microneedles are constructed of a variety of polymers that are biocompatible and biodegradable and are easy to install into the ocular tissue, where they then dissolve upon ocular application and initiate drug release.

Microneedles are structures of a metallic or polymeric nature that range in size from a few to 200 µm. Microneedles contain tiny protrusions, which reduces their degree of invasion. There are several microneedle subtypes with a variety of pharmaceutical purposes; however, just three microneedle subtypes play a substantial role on drug delivery to ocular tissues. These subtypes include solid coated, hollow, and microneedles of dissolving polymers, as shown in Figure 6 [444].

Microneedles with solid coatings are the type that can be used to pierce tissue and the coating instantly disintegrates. They can then be removed. The perforation will create a channel with a diameter of a few microns that will effectively distribute the drug [445]. Consequently, the main goal of solid microneedles is to increase the porosity of the cornea or sclera of the eye (Figure 6a). Metals such as stainless steel and materials such as silicon probes are employed in the manufacture of microneedles. These materials’ non-biodegradability and complexity in production make them undesirable for use in ocular delivery [446]. Coated microneedles have been used successfully to improve the effectiveness of loaded medications in a variety of eye conditions. Pilocarpine, a medication used to treat glaucoma, has shown improved absorption when loaded onto coated microneedles [447]. The anti-VEGF drug, bevacizumab, provided customized medicament delivery to the corneal stroma and a potential impact with fewer adverse effects to treat corneal neovascularization when loaded into coated microneedles [448].

Micron-sized hollow microneedles are needles with the drug formulation entirely inside the needles. The loading of microneedles with drug delivery systems could potentially improve therapeutic drug activity [449]. Microneedles were efficiently loaded with nanoparticles, nano-emulsions, liposomes, and microparticles [450]. Hollow microneedles are mainly composed of borosilicate; however, stainless steel might be used for their manufacturing. The method for administering medication involves puncturing the ocular tissue, after which the drug will leak from the microneedles’ hollow spaces (Figure 6b) [449]. Several medicaments were included in hollow microneedles with improved therapeutic activity. Triamcinolone acetonide was injected into the suprachoroidal area in hollow microneedles to efficiently manage posterior acute uveitis. The utilization of the microneedles helped to successfully alleviate posterior uveitis for up to three days with minimal invasion and without affecting the retina or raising the intraocular pressure [451].

Dissolving polymeric microneedles have been developed as a solution to the many drawbacks of hollow and solid coated microneedles, such as their manufacture, application, and reliability. They have demonstrated their compatibility with ocular tissue in comparison to their hollow and solid coated counterparts [452]. They are made of several biocompatible and biodegradable polymers that are simple to implant into the ocular tissue. After applying the polymeric microneedles to the eye tissue, the medication, which has already been loaded into the polymeric matrix, is released into the eye tissue (Figure 6c) [453]. Dissolving polymeric microneedles have significantly improved the therapeutic action of several drugs. The poorly soluble medication amphotericin B was effectively used with dissolving polymeric microneedles to increase its antifungal effectiveness by enabling rapid dissolution, excellent tissue penetration, low toxicity, and long-lasting therapeutic action [454]. By incorporating cyclosporin A into polymeric microneedles that dissolve, a high molecular weight medication with weak water solubility was effectively delivered to the ocular tissue with improved drug permeability and activity [455].

### 4.3. Three-Dimensional Printable Systems

In recent years, experts have predicted that 3D printing will revolutionize the pharmaceutical industry since it can generate specific doses of individualized medications with novel designs [456], drug mixtures [457], and targeted drug release properties [458]. Additionally, 3D printing could be employed for the development of highly accurate, individualized medical instruments [459]. Over the past 10 years, 3D printing has been heavily utilized in the fields of contact lens manufacturing, drug delivery to ocular tissues, implants, ocular research, and diagnostic models production [460].

Ocular prostheses, which aid ophthalmic patients in restoring the symmetry of their face, were successfully developed throughout 3D bioprinting technology with minimal cytotoxic effects. The 3D-printed prosthesis showed no negative effects on the conjunctival sac or membrane and provided the best resemblance to the look of a human eye, including iris color, sclera, and vascular structures [461]. Ocuserts made by 3D printing were also used to modulate the pharmacokinetics of ganciclovir-loaded glycerosomes, resulting in prolonged release, enhanced tissue penetration, and therapeutic potential [272].

Three-dimensional (3D) printing was additionally incorporated into the development of prosthetic corneal structures in an effort to bypass religious restrictions and drug histories. Artificial corneas created by 3D printing technology were proven a reliable, quick, convenient, and useful choice [462,463]. Gelatin, collagen, polyvinyl alcohol, and sodium alginate are the primary materials used to create the 3D-engineered corneas because they are biodegradable, translucent, permeable to oxygen and nutrients, able to endure shear stress, and sufficiently robust mechanically [464].

The development of artificial retinas is essential for the design of more efficient systems for drug delivery, research into disease causes, and the development of cutting-edge therapeutic choices. Artificial retinas with the best cytocompatibility were created via 3D bioprinting, simulating the natural structure of the human retina [465]. Moreover, human retinal progenitor cells were effectively maintained in vitro by the use of 3D-printed polymeric scaffolds. The subretinal implantation of the cell-free scaffolds into retinitis pigmentosa porcine models did not result in inflammation, infections, or cytotoxicity, supporting the possibility that they may be used in preclinical studies [466].

Dexamethasone-loaded punctal plugs created by 3D printing demonstrated sustained drug release for 1 to 3 weeks, depending on the particular polymer or blends of polymers chosen [37]. Ocular 3D-printed patches have successfully been designed to hold various pharmacological active constituents, and they may be adjusted to release varying amounts based on the patient’s demands [467]. Timolol maleate-loaded 3D-printed contact lenses were successfully utilized to treat glaucoma in patients who did not take their prescribed glaucoma medications [468]. The lenses had a smooth surface with high printing quality and released timolol maleate steadily over a period of three days [468].

Three-dimensional micro-stereolithography has been enrolled in the production of therapeutic devices for controlling intraocular pressure and, consequently, glaucoma. It combines the advantages of both digital light processing and stereolithography technologies. Over the past ten years, minimally invasive glaucoma devices have been designed to boost aqueous humor discharge in an effort to control glaucoma [469]. With the use of 3D printing techniques, a complex surgical device can be produced with significant flexibility while maintaining functionality [470].

### 4.4. In Situ Gelling Implants

Drug implants that are generated when certain conditions are fulfilled are known as in situ forming implants. They are currently quite popular since they do not require regular injections into the eye or insertion via surgery. In situ forming implants are administered as low viscosity solutions that solidify as depots or implants at the targeted site, controlling the administration of drugs [471]. According to the underlying phase separation process, numerous parameters might be used to influence the sol-to-gel transition [472]. In situ gelling implants are advantageous since they are simple to use, very stable, have an optimized drug release profile, and do not require complicated equipment for ocular injection [191].

In situ gelling implants were used to formulate a number of ocularly administered drugs in an effort to increase their therapeutic action, facilitate administration, extend disease management, and boost patient compliance. Moreover, in situ gelling may offer a good substitute for the currently available therapies. Triamcinolone acetonide was successfully developed as a gelling implant with extended drug release that met acceptable rheological and syringe ability standards. Triamcinolone acetonide was maintained by the formulation for a course of six weeks [473]. Bimatoprost was effectively combined into in situ gelling implant for subconjunctival injection with higher stability, cheap cost, improved solubility, and ease of processing using nano-vesicular systems. With just one injection, the newly developed formulation may maintain intraocular pressure for up to 8 weeks [191]. Additionally, peptides were loaded onto polymeric nanoparticles in an effort to lessen the burst release. In situ (light-sensitive) gelling implants with the peptide-loaded nanoparticles were used to deliver the medication to the posterior eye in a sustained and effective manner [474,475].

There are currently no in situ gelling implants available for use in the eyes. However, two formulations, including bevacizumab, are in the final stages of approval. The first is a photosensitive in situ gelling implant (OcuLief^TM^), while the second is a premade photosensitive implant (EyeLief^TM^). Both of these medicines were developed by the Re-Vana Therapeutics corporation [476].

A list of the commercially available long-acting drug delivery systems and devices is shown in Table 2.

## 5. Implantable Systems/Devices for Drug Delivery

Many chronic ocular illnesses necessitate the use of implanted drug-delivery systems or devices (IDDS) for management or therapy. IDDS are made to be implanted in order to regulate the drug efflux and, as a result, lengthen the time that the disease condition is under control. IDDS have significant benefits over conventional systemic administration. Higher medicament concentrations in the intended locations can be achieved via site-specific implantation, which can avoid oral absorption and distribution phases [477]. Additionally, IDDS increases patient compliance, minimizes parenteral treatment pain, and sustains the drug concentration in the therapeutic window by a continuous controlled release of the loaded medication [478]. As a result, IDDS were successfully used in the production of a number of authorized marketed medications to control a variety of chronic diseases, including eye chronic disorders, which include glaucoma, uveitis, endophthalmitis, dry eye diseases, AMD, and diabetic retinopathy. These products include Ozurdex^®^ (Allergan Co., Ltd.), Retisert^®^ (Bausch&Lomb), Vitrasert^®^ (Bausch&Lomb), and I-vation^®^ (Surmodics Inc.). The technologies or techniques used to generate IDDS and characterize these products are discussed in the following sections.

### 5.1. Polymers Used to Formulate IDDS

The choice of polymer is essential for adjusting the release profile of IDDS. Polymers used for intraocular IDDS might be biodegradable or nonbiodegradable. In the next section, we will discuss the polymers often used to formulate ocular IDDS.

#### 5.1.1. Nonbiodegradable Polymers

The virtue of nonbiodegradable polymers, which are used to formulate nonbiodegradable IDDS, is that they may achieve very long-term release and have high biocompatibility [479]. On the other hand, the matrix polymer needs to be surgically removed after drug exhaustion. These polymers include EVA, polyimide, polyethylene terephthalate, and silicones. Several intraocular IDDS are commercially available, including Retisert^®^, Vitrasert^®^, Iluvien^®^, and Renexus^®^.

#### 5.1.2. Biodegradable Polymers

Biodegradable polymers have the benefits of degrading once implanted into biological tissues. However, the type of polymer and degree of crosslinking greatly affect the degradation dynamics [421]. Biodegradable polymers include PLGA, polycaprolactone, and acetyl triethyl citrate. These polymers were successfully employed to develop several commercially available implants, including Ozurdex^®^, Posurdex^®^, Durysta^®^, and Dexycu^®^.

Table 2 outlines several commercially available IDDS with their polymeric composition.

### 5.2. Techniques for the Preparation of IDDS

#### 5.2.1. Solvent Casting

**Figure 7:**
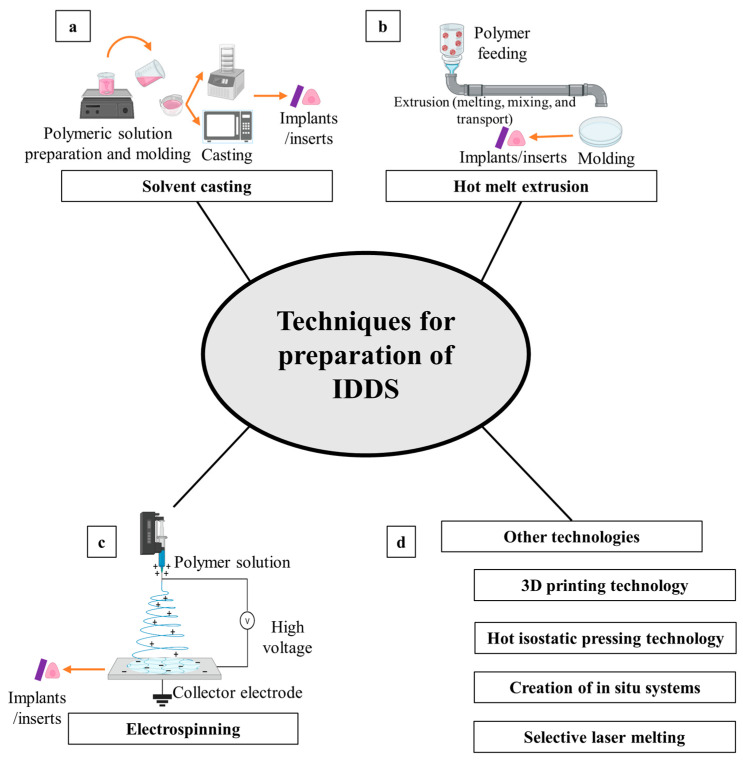
Different techniques/technologies used to generate implantable drug delivery systems. Implantable drug delivery systems might be generated via the use of (**a**) solvent casting, (**b**) melt extrusion, (**c**) electrospinning, and (**d**) other techniques.

For the production of polymeric inserts and implants, solvent casting is an efficient and scalable technique. Various experimental conditions, such as heating and lyophilization, were used to produce and cast polymeric solutions containing drug(s) and plasticizer(s). The type of drug loaded, as well as its thermal stability, play a major role in the choice of the condition. In an effort to improve the stability and therapeutic activity and prolong residency, this approach was used to formulate inserts or implants for several therapeutic medications that had received approval for ocular use. These drugs include dexamethasone [480], acetazolamide [234], bimatoprost [387], etoposide [481], and dorzolamide [482]. Table 3 displays a list of the FDA-approved polymers or copolymers used in ocular preparations. Figure 7a displays a schematic illustration showing the solvent casting process.

#### 5.2.2. Extrusion

The hot melt extrusion method involves forcing a polymer(s) through a mold after it melts or softens at higher temperatures, often with the use of a conveyor system that leads into a tube. The actual process may be split into a number of processes, including heating the polymer mixture, loading, blending, transporting, allowing it to flow through the die, and downstream material processing (Figure 7b) [483]. Controlling each of these stages will ultimately affect the final features of the product [484]. In the hot melting extrusion method, a number of polymers were used, including aliphatic polyesters, poly (ortho esters), polyurethanes, polyvinyl lactams, ethylene-co-vinyl acetate, polyanhydrides, polyacrylics, polyethylene glycol, and polyethylene oxide [483]. In addition, several FDA-approved ocular inserts or implants were developed using the hot melt extrusion technique, including Lacrisert^®^ and Ozurdex^®^ [411,424].

#### 5.2.3. Electrospinning

Electrospun inserts and implants are automatically generated utilizing a system that includes a syringe pump, collector electrode, and high voltage generator (Figure 7c). Several factors may have an impact on the manufactured inserts or implants, including the polymeric solution pump rate, the distance between the syringe tip and collection electrode, and the applied volage [485]. Electrospinning became popular due to its benefits, including simple control of the shape, diameter, surface properties, and porosity, and the simplicity of achieving nanosized inserts/implants [486]. Moreover, electrospinning enables the administration of many medicaments at once.

#### 5.2.4. Other Techniques

Several other techniques might be employed in the development of inserts or implants, including 3D printing, hot isostatic pressing, selective laser melting, and the creation of in situ systems.

Over the past 10 years, printing throughout three dimensions has been frequently employed in the production of implants or inserts [487]. Three-dimensional printing includes the development of inserts or implants by polymer deposition in a layer-by-layer manner [488]. The pharmaceutical industry has lately boosted its usage of 3D printing due to its capacity to produce unique, individualized, and complicated dosage forms and medical equipment [489,490]. A triamcinolone acetonide sustained release implant with great clinical promise was produced as a result of a successful 3D printing application [491]. Further, 3D technology makes it possible to manage the features of the produced implants, including their form, size, and dosage, and to provide customization based on the patient’s clinical situations [491]. Hot melt extrusion coupled with 3D-printed fused deposition were effectively used to generate ciprofloxacin-loaded ocular inserts that have improved therapeutic results for treating ocular pathogenic infections and sustained antibacterial activity [492].

In the industrial process known as hot isostatic pressing, components or powders are heated to a high temperature while also being compressed in a pressurized cylinder [493]. Metal-based implants made of titanium [494] and stainless steel [495] are frequently produced via hot isostatic pressing.

Selective laser melting primarily relies on the employment of a high intensity laser beam to fuse the powder that is present in its focus zone and enable the manufacturing of items layer-by-layer from a 3D computer-assisted design [496]. The production of inserts or implants is now regarded to be a viable application for selective laser melting [496]. Selective laser melting makes it feasible to generate implants that have a crooked structure, which was previously not conceivable commercially.

In situ forming implants are solutions that go through phase separation to produce a drug depot formulation. Crosslinking, solidifying, and phase separation are some of the in situ gelling systems’ mechanisms [472]. In situ crosslinking of polymers could be initiated chemically, physically, or through photosensitization, while solidifying organogels initiate in situ gelling through solubility alteration. In situ gelling through phase separation systems could be triggered by a pH change, temperature change, or solvent exchange [472]. Drug-loaded in situ ocular inserts or implants provide benefits in terms of better therapeutic action, increased stability, simplicity of administration, and control over drug release [191,497].

### 5.3. Characterization and Evaluation of IDDS

In vitro testing and characterization of drug delivery systems or devices is a key element in pharmaceutical development’s quality control process for evaluating and determining the best formulation(s). One of the most crucial characterization criteria, in vitro testing for dissolution, is utilized to develop in vitro–in vivo relationships, which aid formulation marketing and reduce medication costs. However, the idea of developing in vitro–in vivo correlations becomes more difficult for IDDS, owing to the sophistication of ocular physiological conditions, ocular tissue barriers, and the insertion site of the implant. Therefore, the design of practical in vitro testing for drug release and dissolution from IDDS remains challenging. Several in vitro simulation experiments have been developed to mimic the in vivo insertion of IDDS in the eye tissue.

**Figure 8:**
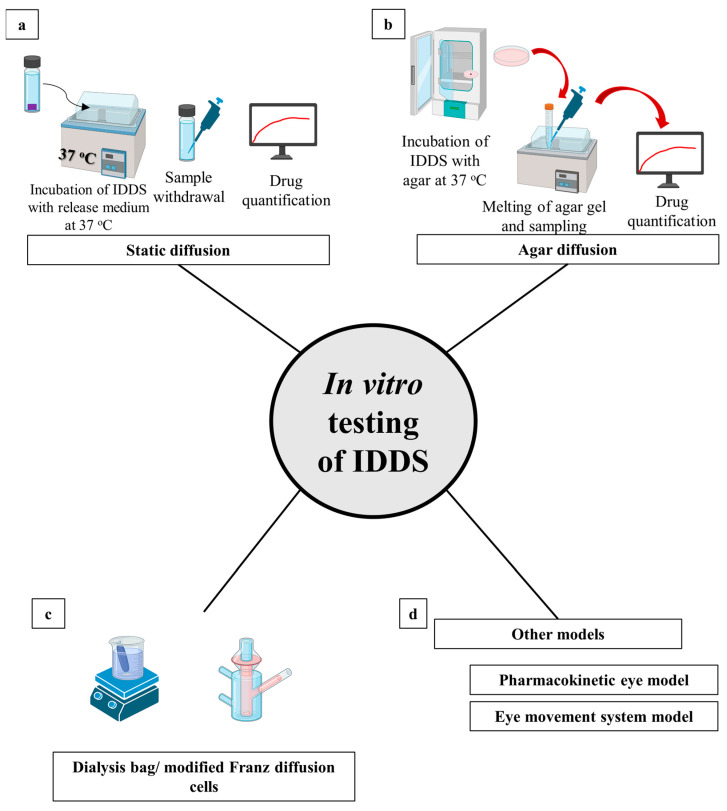
In vitro testing of implantable drug delivery systems. Implantable drug delivery systems might be characterized for drug dissolution and release using (**a**) static diffusion, (**b**) agar diffusion, (**c**) dialysis bags, and (**d**) other models.

The static diffusion system (Figure 8a) was developed to investigate the in vitro efflux rate of drugs formulated as IDDS. In this system, the appropriate release media is chosen and directly incubated with the IDDS and kept at a standard temperature with or without mechanical agitation [498]. The amount of medication released is then measured at predetermined intervals of time. Despite the static diffusion method’s widespread use, it was restricted in its capacity to investigate IDDS because it lacked ocular flow modelling and the capacity to control diffusion layers [499].

The agar diffusion system was also adopted to assess the drug release from IDDS under very viscous conditions [500]. The procedures involved inserting IDDS into agar gel and, using the proper analytical technique, the gel was evaluated for the amount of drug dispersed at predetermined time intervals [500]. Figure 8b displays a schematic illustration of the procedures involved. However, the implants are made to be inserted in certain environments; this technique does not accurately reflect such environments. Additionally, this method only relies on a diffusion mechanism to control the drug outflow from the implant, avoiding any potential impact from the actual vitreous environment [499].

The dialysis bags system is the most straightforward method for predicting the in vitro dissolution and release of therapeutic drugs from IDDS. This technique employed a dialysis bag, which was closed on both sides once the implant was inserted (Figure 8c). Drug molecules should be able to pass through the specified molecular weight cutoff for the dialysis bag. After that, the dialysis bag is placed in the release medium solution, which is constantly agitated at standard temperature. At regular intervals, samples from the release media were obtained, analyzed, and quantified [501]. The Franz diffusion cells or modified Franz diffusion cells with a modified curved donor compartment to accommodate the curvature of the excised corneal tissues operates with the same principle of the dialysis bag, but with a more consistent and reproducible surface area for drug diffusion; they have the capacity to hold ocular tissues.

The pharmacokinetic eye model is a more complex system that simulates drug clearance via the anterior chamber, including intraocular aqueous outflow. The apparatus has two compartments that are partitioned by a dialysis membrane that simulates the posterior and anterior ocular chambers. It is hypothesized that this model may also be used to determine how much of the drug would be released from IDDS that are placed in the cavity of the vitreous [502]. The device was designed to mimic the actual insertion operation of IDDS into the eye. Both the injection and the aqueous inflow ports were positioned inside the replicating vitreous cavity, while a single output port was positioned in the simulating anterior chamber. The device proved highly effective in evaluating the in vitro release studies of various commercially available medicines, including Kenalog^®^, Avastin^®^, and Lucentis^®^.

The eye movement system model was created as an in vitro simulation system to imitate the vitreous body, as well as environmental stimuli that move the eyes, such as head movement [503]. IDDS were inserted inside the chamber, which imitates eye and head movements. The release medium is refreshed every 24 h, and the drug concentration is assessed using the proper analytical technique. The method may demonstrate how the vitreous body’s gelled region, together with conscious motions of the head and eyes, influence the release of produced IDDS.

Despite the wide advancements of in vitro testing, there is still no in vitro experimental design that accurately mimics the factors that determine release in an in vivo setting. This happens as a result of the drug’s distribution and penetration process in the eye being more difficult to simulate than with other routes. Furthermore, it would be unethical to repeatedly monitor drug levels in a living eye in order to demonstrate an in vitro–in vivo association relationship.

### 5.4. Sites for Delivery and Implantation

The selection of an implant location is contingent on the required pharmacokinetics, biocompatibility, and clinical factors. A close proximity between the insertion site and the target tissue allows for a high drug concentration in the target tissue. The most popular sites for implantation include intravitreal [419], intracameral [420], and subconjunctival injections [504]. The intravitreal injection is widely used to deliver several commercially available corticosteroids, anti-VEGF agents, antivirals, and encapsulated cells (Table 2). Extensive research was conducted on intracameral injection during the past decade, and as a consequence, the FDA granted approval to the first intracamerally injected implant, Durysta, in the year 2020 (Table 2) [505]. Subconjunctival injection is considered one of the most effective approaches to deliver several medications to the vitreous and retinal area at higher levels [506].

### 5.5. Regulatory Aspects of IDDS

The FDA classifies IDDS as either class II or class III medical devices, which, respectively, denote intermediate and notably higher risk levels, because of their direct and persistent contact with the living tissues [507]. In order for IDDS to be approved by the FDA and marketed in the USA, it needs to obtain the pre-market notification 510(K). A 510(K) is a premarket application submitted to the FDA to prove that the product being marketed is essentially identical to, or equally safe and effective to, a product that has previously gained FDA approval [508]. If there is no comparable product on the market, the innovative device must receive pre-market approval with sufficient reliable scientific data that must prove that it is effective and safe for the intended usage(s) [509].

For medical device manufacturers to follow guidelines while designing, producing, packing, and distributing their products, the FDA introduced “Design Control Guidance for Medical Device Manufacturers”. The FDA periodically inspects manufacturers to ensure that they adhere to the required good manufacturing practice requirements [510]. For IDDS approval, further laboratory tests, including those for sterility, biocompatibility, and material characterization, are required.

Sterilization assures patient safety during implantation procedures via the lack of live microorganisms on the device. The FDA recommends terminal sterilization using either ethylene oxide or gamma radiation. A vital component of good terminal sterilization is the packaging mechanism, which must permit gas penetration and radiation to reach the biomaterial. The FDA’s primary criteria are equipment validation, microbiological testing, and sterilization testing [511].

The chosen material must also be biocompatible and should not result in any undesirable unfavorable biological reactions when in contact with the human body. The material’s biocompatibility must be verified with tests for cytotoxicity, hemocompatibility, pyrogenicity, sensitization, genotoxicity, and carcinogenicity [511].

The physical, chemical, and mechanical characteristics should be determined for biomaterials allowed to generate IDDS. The pore size, pore size distribution, structure, and connectivity are examples of physical characteristics. The potential for toxicity, carcinogenicity, and immunogenicity are all factors of chemical characterization, and compressibility and mechanical strength are examples of mechanical properties [512].

## 6. Conclusions and Future Prospective

The use of IDDS is advantageous for the management of a number of ocular chronic disorders, including glaucoma, uveitis, endophthalmitis, and retinal disease, over the traditional ocular dosage forms. Less frequent administration, sustained and local action, bypassing several ocular barriers, and prolonged pharmacological impact are some of the positive characteristics of IDDS [386]. However, IDDS suffer several limitations that affect their pharmacological activity. IDDS are considered an invasive technique for ocular drug delivery. Additionally, some IDDS that are not biodegradable need to be surgically removed at the end of the treatment period, which has an impact on patient compliance. Likewise, IDDS were made to release the loaded drug over the course of treatment at a fixed value without being affected by environmental factors. A change in the medication release profile could be necessary, though, due to fluctuations in the course of the disease, how it responds to therapy, and other disorders. The key obstacle to the successful implementation of adjustable delivery ocular implantable drug delivery systems/devices is still related to their size limitation, which necessitates the use of extremely potent medications to accomplish long-term release. While this succeeds well with most steroids, it may cause issues with certain larger biomolecules. Additionally, the expense of therapy is greatly increased by injection and retrieval procedures for currently marketed IDDS.

This review has identified the most prevalent ocular chronic disorders that require longer treatment durations with their therapeutic drugs and the most advanced systems for drug delivery, which might be able to boost the activity, stability, and penetration of these pharmaceuticals throughout the ocular tissue. The enrollment of drugs into advanced systems for drug delivery may be sufficient to surmount all the impediments that stand in the way of drug activity. This approach is also more cost-effective than creating more effective drug molecules with desirable properties. This review also concentrated on the use of long-acting drug delivery systems, particularly IDDS, and their production processes, techniques for characterization, and assessment, as well as the legal and ethical issues of their clinical implication.

## Figures and Tables

**Table 2:** Commercial ocular drug delivery systems/devices for different chronic eye conditions.

Platform/Device	Commercial Brand	Therapeutic Agent (Approval Year, Country)	Excipient Composition	Clinical Implication	Route of Administration	Refs.
Liposomes	Visudyne^®^	Verteporfin (2000, USA)	Dimyristoylphosphatidylcholine and egg yolk phosphatidylglycerol	Choroidal neovascularization in AMD	IV	[393]
	Amphotec^®^	Amphotericin B (1996, USA)	Cholesteryl sulfate	Fungal endophthalmitis	IV	[394]
	Abelcet^®^	Amphotericin B (1995, USA)	Dimyristoylphosphatidylcholine and dimyristoylphosphatidylglycerol	Fungal endophthalmitis	IV	[395]
	AmBisome^®^	Amphotericin B (1997, USA)	Hydrogenated soy phosphatidylcholine, cholesterol, and distearoylphosphatidylglycerol	Fungal endophthalmitis	IV	[396]
	Ozodrop^®^	Sunflower ozonized oil (NA, Italy)	LipozonEye, hypromellose and polyhexamethylene biguanide	Post-cataract surgery inflammation	Topical	[397]
	Lacrisek^®^	Vitamin A palmitate, vitamin E (NA, Italy)	Hydrogenated phospholipids	Dry eye syndrome	Topical	[398]
	Tears Again^®^	Vitamin A palmitate, vitamin E (NA, USA)	Soy lecithin, phenoxyethanol	Dry eye syndrome	Topical	[398]
Emulsions	Restasis^®^	Cyclosporine A (2002, USA)	Polysorbate 80, castor oil	Dry eye syndrome	Topical	[399]
	Emustil^®^	Cyclosporine A (NA, Italy)	Soybean oil, egg yolk phospholipids	Dry eye syndrome	Topical	[400]
	Refresh Endura^®^	Cyclosporine A (2020, USA)	Polysorbate 80, castor oil	Dry eye syndrome	Topical	[401]
	Xelpros^®^	Latanoprost (2018, USA)	Castor oil, propylene glycol	Glaucoma	Topical	[402]
	Durezol^®^	Difluprednate (2008, USA)	Castor oil, polysorbate 80	Diabetic macular edema	Topical	[85]
	Verkazia^®^	Cyclosporine A (2017, UK)	Medium-chain triglyceride, Tyloxapol, and poloxamer 188	Vernal keratoconjunctivitis	Topical	[403]
Micelles	Cequa^®^	Cyclosporin A (2018, USA)	Polyoxyl hydrogenated castor oil, polyalkoxylated alcohol	Dry eye syndrome	Topical	[54]
	AzaSite^®^	Azithromycin (2007, USA)	Polycarbophil	Blepharitis	Topical	[404]
Micro and nanoparticles	Macugen^®^	Pegaptanib (2004, USA)	PEG-40kDa	AMD	IVT	[405]
	Trivaris^®^	Triamcinolone acetonide (2005, USA)	Sodium hyaluronate	Uveitis	IVT	[406]
	Inveltys^®^	Loteprednol etabonate (2018, USA)	Pluronic F127	Post-operative inflammations	Topical	[407]
	Eysuvis^®^	Loteprednol etabonate (2020, USA)	Pluronic F127	Dry eye syndrome	Topical	[407]
	Triesence^®^	Triamcinolone acetonide (2005, USA)	Carboxymethyl cellulose	Dry eye syndrome	IVT	[408]
	Tobradex ST^®^	Tobramycin and dexamethasone (2003, USA)	Xanthan gum	Bacterial conjunctivitis	Topical	[403]
	BromSite^®^	Bromfenac (2016, USA)	Polycarbophil	Post-operative inflammation and pain reliver	Topical	[409]
	Besivance^®^	Besifloxacin (2009, USA)	Polycarbophil	Bacterial conjunctivitis	Topical	[410]
Implants	Ozurdex^®^	Dexamethasone (2009, USA)	Acid-terminated PLGA (30%) + ester-terminated PLGA (10%)	Macular edema	IVT	[411]
	Retisert^®^	Fluocinolone acetonide (2005, USA)	Ethylene-vinyl acetate coated with polyvinyl alcohol	Uveitis	IVT	[412]
	Vitrasert^®^	Ganciclovir (1996, USA)	Ethylene-vinyl acetate coated with polyvinyl alcohol	Cytomegalovirus retinitis	IVT	[412]
	I-vation^®^	Triamcinolone acetonide (2007, USA)	Poly (methyl methacrylate) and ethylene vinyl acetate	Diabetic macular edema	IVT	[413]
	Iluvien^®^	Fluocinolone acetonide (2014, USA)	Polyimide tube coated with Polyvinyl alcohol	Diabetic macular edema	IVT	[414]
	Medidur^®^	Fluocinolone acetonide (2014, USA)	Polyvinyl alcohol	Diabetic macular edema	IVT	[415]
	Posurdex^®^	Dexamethasone (2009, USA)	PLGA	Macular edema	IVT	[416]
	Surodex^®^	Dexamethasone (2008, USA)	PLGA	Post-operative inflammation	Subscleral placement	[417]
	Renexus^®^	Encapsulated cell technology (NA)	Polyethylene terephthalate	AMD	IVT	[418]
	Yutiq^®^	Fluocinolone acetonide (2018, USA)	Polyimide/polyvinyl alcohol	Diabetic macular edema	IVT	[419]
	Durysta^®^	Bimatoprost (2020, USA)	Poly(D,L-lactide), PLGA, and poly (D,L-lactide) with an acid end group	Glaucoma	ICI	[420]
	Dexycu^®^	Dexamethasone (2018, USA)	Acetyl triethyl citrate	Post-operative inflammation	Posterior chamber injection	[421]
	Susvimo^®^	Ranibizumab (2020, Germany)	Polysulphone, silicone	AMD	IVT	[422]
Inserts	Ocusert^®^ *	Pilocarpine (1972, USA)	Polyethylene co-vinyl acetate	Glaucoma	CI	[423]
	Lacrisert^®^	Hydroxypropyl methyl cellulose (1992, USA)	Hydroxypropyl methyl cellulose	Moderate-to-severe dry eye syndrome	CI	[424]
	BIM ring^®^	Bimatoprost (NA)	Support made of polypropylene and covered in a silicone matrix.	Glaucoma	CI	[425,426]
	Dextenza^®^	Dexamethasone (2018, USA)	Polyethylene glycol	Post-operative inflammation	CI	[414]
	Mydriasert^®^	Tropicamid, phenylephrine hydrochloride, and hydroxypropyl methyl cellulose (2015, UK)	Ammonium methacrylate copolymer	Diagnosis (pupil dilator)	Intracanalicular insertion	[427]
Microneedles	Xipere™	Triamcinolone acetonide (2019, USA)	Carboxymethylcellulose sodium, and polysorbate 80	Macular edema associated with uveitis	SCS	[428,429]
Drug eluting contact lens	Acuvue^®^	Ketotifen fumarate (2017, USA)	Etafilcon A	Ocular allergic itch	Topical	[430]

Abbreviations: IVT: intravitreal injection; IV: intravenous; PLGA: poly (lactic-co-glycolic acid); SCS: suprachoroidal space injection; ICI: intracameral injections; CI: cul-de-sac insertion. * The pilocarpine-loaded non-biodegradable insert (Ocusert^®^) is obsolete due to lack of clinical outcomes.

**Table 3:** List of polymers that the FDA has authorized for use in the manufacture of ocular formulations, (https://www.accessdata.fda.gov/scripts/cder/iig/index.cfm; accessed on 25 September 2022).

Polymer	Route	Pharmaceutical Forms	CAS Number
Carbomer a	Eye surface	Emulsion	
Carbomer b	Eye surface	Emulsion	
Carbomer b	Eye surface	Gel	
Carbomer b	Eye surface	Suspension	
Carbomer b	Eye surface	Suspension/drops	
Carbomer b	Eye surface	Suspension	
Carbomer b	Eye surface	Suspension/drops	
Carbomer c	Eye surface	Gel	
Ethylene-vinyl acetate copolymers (EVA)	Eye surface	Insert, extended release	24937788
Ethylene-vinyl acetate copolymers (EVA)	Eye surface	Solution	24937788
PEG/PPG-4/30 copolymer	Eye surface	Solution	
PLGA	Intravitreal	Implant	26780507
PLGA	Intravitreal	Injection	26780507

## Data Availability

The data are available from the corresponding author upon request.
